# Prognostic factors in patients with submucosal carcinoma of the oesophagus

**DOI:** 10.1054/bjoc.2000.1288

**Published:** 2000-08-16

**Authors:** M Watanabe, H Kuwano, K Araki, H Kawaguchi, H Saeki, K Kitamura, S Ohno, K Sugimachi

**Affiliations:** The Department of Surgery II, Faculty of Medicine, Kyushu University, Maidashi, Higashi-ku, Fukuoka, 812, Japan; the Department of Surgery I, Faculty of Medicine, Gunma University, Showa-machi, Maebashi, Gunma, 371, Japan

**Keywords:** submucosal carcinoma of the oesophagus, vessel invasion, intramural metastasis, lymph node metastasis

## Abstract

To clarify the prognostic factors in patients with submucosal carcinoma of the oesophagus, we examined the results of surgical treatment for 78 cases over the last decade. The clinicopathological factors including age, sex, location of the tumour, length of the tumour, histological differentiation, subclassification of depth, lymphatic or blood vessel invasion, intramural metastasis and lymph node metastasis were all analysed. Then the correlation between these factors and prognosis was investigated. As a result, significant differences were observed in the survival rates between the groups regarding lymphatic vessel invasion (*P* = 0.0003), intramural metastasis (*P* = 0.0051) and lymph node metastasis (*P* = 0.0026). According to a multivariate analysis, intramural metastasis (*P* = 0.0038, relative risk 9.17), vessel invasion (*P* = 0.0033, relative risk 6.25) and lymph node metastasis (*P* = 0.0187, relative risk 3.62) were found to be independent prognostic factors. The prognosis of the patients with at least one of these factors was significantly poorer than that without. The five-year survival rate of the patients without these factors was as good as that with mucosal carcinoma of the oesophagus. Based on our findings, vessel invasion, intramural metastasis and lymph node metastasis are thus considered to be significant prognostic factors in patients with submucosal carcinoma of the oesophagus. © 2000 Cancer Research Campaign


British Journal of Cancer (2000) 83(5), 609–613
© 2000 Cancer Research Campaign
doi: 10.1054/ bjoc.2000.1288, available online at http://www.idealibrary.com on IDEAL®

### Prognostic factors in patients with submucosal carcinoma of the oesophagus

**M Watanabe[1], H Kuwano[2], K Araki[1], H Kawaguchi[1], H Saeki[1], K Kitamura[1], S Ohno[1], and K Sugimachi[1]**

[1]The Department of Surgery II, Faculty of Medicine, Kyushu University, Maidashi, Higashi-ku, Fukuoka 812, Japan and [2]the Department of Surgery I, Faculty of Medicine, Gunma University, Showa-machi, Maebashi, Gunma 371, Japan

**Summary** To clarify the prognostic factors in patients with submucosal carcinoma of the oesophagus, we examined the results of surgical treatment for 78 cases over the last decade. The clinicopathological factors including age, sex, location of the tumour, length of the tumour, histological differentiation, subclassification of depth, lymphatic or blood vessel invasion, intramural metastasis and lymph node metastasis were all analysed. Then the correlation between these factors and prognosis was investigated. As a result, significant differences were observed in the survival rates between the groups regarding lymphatic vessel invasion ($P$ = 0.0003), intramural metastasis ($P$ = 0.0051) and lymph node metastasis ($P$ = 0.0026). According to a multivariate analysis, intramural metastasis ($P$ = 0.0038, relative risk 9.17), vessel invasion ($P$ = 0.0033, relative risk 6.25) and lymph node metastasis ($P$ = 0.0187, relative risk 3.62) were found to be independent prognostic factors. The prognosis of the patients with at least one of these factors was significantly poorer than that without. The five-year survival rate of the patients without these factors was as good as that with mucosal carcinoma of the oesophagus. Based on our findings, vessel invasion, intramural metastasis and lymph node metastasis are thus considered to be significant prognostic factors in patients with submucosal carcinoma of the oesophagus. © 2000 Cancer Research Campaign

**Keywords:** submucosal carcinoma of the oesophagus; vessel invasion; intramural metastasis; lymph node metastasis

Recent advances in diagnostic techniques have facilitated in the early detection of gastrointestinal carcinomas and have also contributed to the improvement of treatment results. Patients presenting with superficial oesophageal carcinoma limited to within the submucosal (sm) layer have also increased in recent years (Yoshinaka et al, 1991; Sugimachi et al, 1993), owing to the popularization of panendoscope and Lugol's solution staining techniques (Brodmerkel, 1971; Sugimachi et al, 1989).

The treatment results of patients with mucosal carcinoma of the oesophagus have been reported to be favourable, and then an endoscopic mucosal resection has been considered to be indicated for epithelial and lamina propria mucosae cancers (Akiyama et al, 1994a; Kodama and Kakegawa, 1998). In contrast, the prognosis of patients with advanced oesophageal cancer is still poor contrary to the efforts such as an extended lymphadenectomy (Isono et al, 1991). Recently, multimodal treatment including chemotherapy, irradiation and hyperthermia has been indicated for cases with locally advanced tumours (Lehnert, 1999).

Although the prognosis of patients with sm carcinoma has been reported to be significantly superior to that with advanced carcinoma, it remains still unsatisfactory (Sugimachi et al, 1991; Kitamura et al, 1993; Nabeya and Nakata, 1997). There have been still more than a few patients who died of recurrent carcinoma, especially due to distant organ metastasis (Watanabe et al, 1995), even when a curative oesophagectomy had been performed. In such cases, the carcinoma should be considered to be a systemic disease at the time of the operation, and that it is impossible to cure them by radical surgery alone. Therefore, it is necessary to select a group of patients who have a high risk of recurrence and to administer an adequate adjuvant therapy. However, only a few attempts have so far been made at identifying types of tumour have a high risk of recurrence.

The aim of this study is to clarify the prognostic factors in patients with sm carcinoma of the oesophagus, using a univariate and multivariate analysis on 78 patients who had been operated on over the past decade.

*Received 22 September 1999*
*Revised 25 February 2000*
*Accepted 13 April 2000*

*Correspondence to:* M Watanabe

### PATIENTS AND METHODS

### Patients

From 1988–1997, 397 patients with oesophageal carcinoma underwent an oesophagectomy at the Department of Surgery II, Kyushu University Hospital. Among these patients, 116 (29.2%) had superficial oesophageal cancer, including 6 intraepithelial, 32 mucosal and 78 sm carcinomas. All 78 patients with sm carcinoma who underwent an oesophagectomy with lymph node dissection were investigated.

### Histological examination

All the resected specimens, including the lymph nodes, were histopathologically examined. Microscopic sections of the entire resected oesophagus were prepared from step-sectioned blocks, measuring 5 mm in width, after staining with haematoxylin and eosin, and such histological features as the depth and extent of the lesions, involvement to either the lymphatic or blood vessels, intramural metastasis and lymph node metastasis were evaluated.

Pathologic diagnosis and classification were made according to Guidelines for the Clinical and Pathologic Studies on Carcinoma of the Esophagus (Japanese Society for Esophageal Diseases, 1999). The depth of penetration was subclassified into 3 grades; namely sm1, sm2 and sm3 (Nabeya and Nakata, 1997). Sm1 is defined as one-third invasion in the superficial submucosal layer, sm2 as one-third invasion in the medium layer, and sm3 as invasion in the deep layer. Lymphatic vessel invasion (LVI) was regarded as definite when cancer cells were detected in the thin-walled endothelium-lined space containing no red blood cells but occasionally containing lymph fluid (Sarbia et al, 1995) (Figure 2A). Blood vessel invasion (BVI) was defined as tumour cells and red blood cells within the round or ovoid endothelium-lined spaces with a smooth muscle layer (Theunissen et al, 1991; Inoue et al, 1992; Sarbia et al, 1995). The presence of an adjacent artery aided in detecting vascular invasion (Inoue et al, 1992) (Figure 2B). Intramural metastasis (IM) was defined as a metastatic tumour within the oesophageal wall which was separate from the primary tumour, not located within a vessel lumen but rather observed to invade the oesophageal stroma and not accompanied by an intraepithelial cancerous lesion (Takubo et al, 1990; Kuwano et al, 1994) (Figure 2C).

### Follow-up

None of the 78 patients died within 30 days of the operation or during their hospital stay. All patients were followed up regularly at our institution or at affiliated hospitals with routine physical and laboratory examination after discharge. Follow-up examinations, such as computed tomography, ultrasonography and upper GI series were carried out every 3 or 6 months. Endoscopic examinations were performed when appropriate. The progress of the patients was monitored for more than 2 years after surgery.

### Statistical analysis

The clinicopathological parameters included age, sex, location of the tumour, length of the tumour, histological differentiation, subclassification of depth, lymphatic invasion, blood vessel invasion, intramural metastasis and lymph node metastasis. The survival rates were calculated by the Kaplan-Meiyer method and statistically significant differences were analysed by the log-rank test. Variables including age, location, length, differentiation, subclassification of depth, vessel invasion (VI), IM and lymph node metastasis (LN) were subjected to a multivariate analysis using a proportional hazards model to identify any independent predictors regarding the prognosis.

### RESULTS

The survival curves of patients with superficial carcinoma of the oesophagus are shown in Figure 1. The three- and five-year survival rates of 78 patients with sm carcinoma were 80.8% and 74.3%, respectively, and were lower than those with either intra-epithelial or mucosal carcinoma (statistically not significant).

The clinicopathologic factors and prognosis for patients with sm carcinoma of the oesophagus are shown in Table 1. No significant difference in the survival rates was observed between the groups divided according to age, sex, location of the tumour, length of the tumour, histological differentiation and subclassification of depth. There were 17 cases (21.8%) with LVI, 7 cases (9.0%) with BVI and 4 cases (5.1%) with IM. LN was recognized in 24 cases (30.8%). Significant differences were observed in the survival rates between the groups with LVI ($P$ = 0.0003), IM ($P$ = 0.0051) and LN ($P$ = 0.0026). Although there was no significant difference between the groups with and without BVI, the three-year survival rate of patients with BVI was no more than 50%. Then we examined the survival curves between the patients with and without VI. As a result, the three- and five-year survival rates of the patients with VI were 50.9% and 45.3%, respectively, while those without VI were 95.9% and 89.3%, respectively. There was a significant difference between these groups ($P$ < 0.0001).

A multivariate analysis on factors including age (≤70), location (upper), length (≤3.6 cm), differentiation (poorly), subclassification of depth (sm3), VI (positive), IM (positive) and LN (positive) was shown in Table 2. As a result, IM ($P$ = 0.0038, relative risk 9.17), VI ($P$ = 0.0033, relative risk 6.25) and LN ($P$ = 0.0187, relative risk 3.62) were found to be significant factors. A total of 15 patients died of recurrent carcinoma after an oesophagectomy for sm carcinoma in this series. The site of recurrence and the state of VI, IM and LN are shown in Table 3. There were 14 of 15 cases (93.3%) with at least one of these factors. These results also indicated the prognostic significance of VI, IM and LN. We next compared the survival curves between the cases with none of VI, IM and LN (Group I) and the others (Group II) (Figure 3A), and between the cases with neither VI nor IM (Group III) and the others (Group IV) (Figure 3B). Significant differences were observed between Groups I and II ($P$ = 0.002), and between Groups III and IV ($P$ < 0.0001). The five-year survival rate of Group I patients was as good as that of mucosal carcinoma of the oesophagus.

### DISCUSSION

The number of patients with superficial carcinoma of the oesophagus has recently been increasing in Japan (Sugimachi et al, 1989; Yoshinaka et al, 1991). In our department, there were 397 patients with oesophageal carcinoma who had undergone an oesophagectomy over the last decade and 116 of them (29.2%) had superficial carcinoma, while there were only 39 of 364 cases (10.7%) before

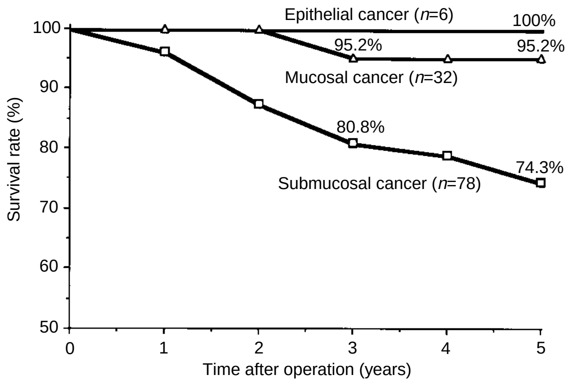

**Figure 1** Survival of the patients with superficial carcinoma of the oesophagus (1988–1997, Department of Surgery II, Kyushu University Hospital).

© 2000 Cancer Research Campaign

**Table 1** Clinicopathological parameters and suvival analysis for patients with submucosal carcinoma of the oesophagus

| Factors | | Cases (%) | Survival rate (%) | | | | | *P* value |
|---|---|---|---|---|---|---|---|---|
| | | | 1 yr | 2 yr | 3 yr | 4 yr | 5 yr | |
| Age | ≤59 | 30 (38.5) | 96.6 | 85.8 | 77.8 | 77.8 | 72.9 | 0.6084 |
| | 60–69 | 30 (38.5) | 96.7 | 89.1 | 85.0 | 85.0 | 85.0 | |
| | ≥70 | 18 (23.0) | 94.4 | 87.2 | 78.5 | 69.7 | 58.1 | |
| Sex | Male | 74 (94.9) | 95.8 | 86.6 | 80.0 | 78.1 | 73.5 | N.S. |
| | Female | 4 (5.1) | 100 | 100 | 100 | 100 | 100 | |
| Location | Upper | 12 (15.4) | 90.9 | 77.9 | 77.9 | 77.9 | 58.4 | 0.8416 |
| | Middle | 41 (52.6) | 97.4 | 89.1 | 83.0 | 79.4 | 75.4 | |
| | Lower | 25 (32.1) | 96.0 | 88.0 | 78.2 | 78.2 | 78.2 | |
| Length | >3.6 cm | 48 (61.5) | 97.9 | 88.3 | 82.8 | 82.8 | 74.7 | 0.6431 |
| | ≤3.6 cm | 30 (38.5) | 93.0 | 85.3 | 77.2 | 72.9 | 72.9 | |
| Differentiation of SCC[a] | Well | 8 (10.3) | 100 | 100 | 100 | 100 | 80.0 | 0.9978 |
| | Mod[b] | 45 (57.7) | 93.1 | 88.0 | 82.6 | 79.3 | 75.1 | |
| | Poor[b] | 20 (25.6) | 100 | 79.2 | 73.5 | 73.5 | 73.5 | |
| | Others | 5 (6.4) | | | | | | |
| Subclassification of depth[c] | sm1 | 15 (19.2) | 93.3 | 93.3 | 84.0 | 84.0 | 84.0 | 0.1627 |
| | sm2 | 27 (34.6) | 96.3 | 91.5 | 91.5 | 91.5 | 84.9 | |
| | sm3 | 36 (46.2) | 97.2 | 82.3 | 72.6 | 69.2 | 64.6 | |
| Lymphatic invasion | Negative | 61 (78.2) | 100 | 94.3 | 90.0 | 87.4 | 84.4 | 0.0003 |
| | Positive | 17 (21.8) | 82.4 | 63.7 | 51.0 | 51.0 | 42.5 | |
| Blood vessel invasion | Negative | 71 (91.0) | 95.7 | 87.7 | 84.1 | 82.0 | 76.8 | 0.1845 |
| | Positive | 7 (9.0) | 100 | 83.3 | 50.0 | 50.0 | 50.0 | |
| Intramural metastasis | Negative | 74 (94.9) | 97.3 | 87.9 | 82.7 | 80.7 | 78.3 | 0.0051 |
| | Positive | 4 (5.1) | 75.0 | 75.0 | 50.0 | 50.0 | 0 | |
| Lymph node metastasis | Negative | 54 (69.2) | 100 | 95.6 | 88.4 | 85.7 | 82.6 | 0.0026 |
| | Positive | 24 (30.8) | 87.1 | 68.9 | 64.0 | 64.0 | 54.8 | |

[a]SCC, Squamous cell carcinoma. [b]Mod, moderately; Poor, poorly. [c]sm1, one-third invasion in the superficial submucosal layer; sm2, one-third invasion in the medium layer; sm3, one-third invasion in the deep layer.

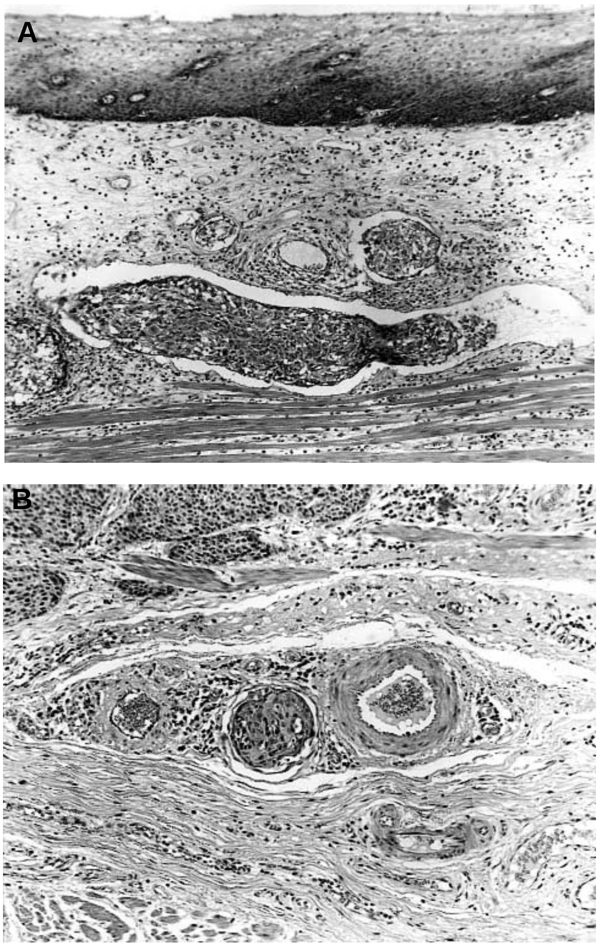

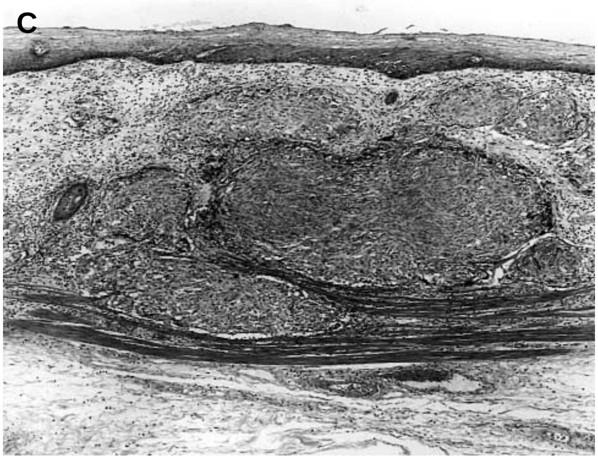

**Figure 2** (**A**) Lymphatic vessel invasion (×70): the cancer cells were detected in the thin walled endothelium-lined space containing no red blood cells but containing lymph fluid. (**B**) Blood vessel invasion (×100): the tumour cells and red blood cells within the round or ovoid endothelium-lined spaces with a smooth muscle layer. An adjacent artery is recognized. (**C**) Intramural metastasis (×38): the metastatic tumour within the esophageal wall which was separate from the primary tumour, and not located within a vessel lumen but rather observed to invade the oesophageal stroma, and not accompanied by an intraepithelial cancerous lesion.

this period (data not shown). Regarding the reason for the increased incidence, many authors have pointed out the recent trends in the frequent use of panendoscopy, especially in asymptomatic patients, and also the use of Lugol's solution staining technique (Shimizu et al, 1983; Schmidt et al, 1986; Bogomoletz et al, 1989; Sugimachi et al, 1989). Cases with sm carcinoma are reported to amount to approximately 70% of all patients with superficial carcinoma of the oesophagus (Yoshinaka et al, 1991).

© 2000 Cancer Research Campaign

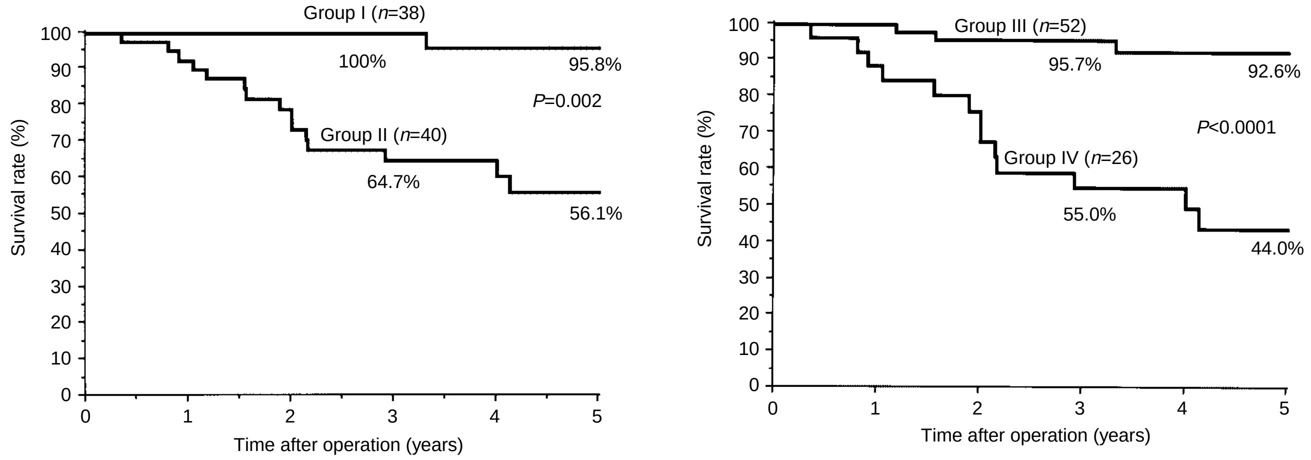

**Figure 3** Survival of the patients with submucosal carcinoma of the oesophagus. (**A**) Group I, patients with none of vessel invasion, intramural metastasis and lymph node metastasis; Group II, patients with at least one of these factors. (**B**) Group III, patients with either vessel invasion nor intramural metastasis; Group IV, patients with either vessel invasion or intramural metastasis. A significant difference was observed in the survival between Groups I and II ($P = 0.002$), and Groups III and IV ($P < 0.0001$).

**Table 2** A multivariate analysis

| Variable | Coefficient | Standard error | Relative risk | *P* value |
|---|---|---|---|---|
| Intramural metastasis (positive) | 2.221 | 0.768 | 9.17 | 0.0038 |
| Vessel invasion (positive) | 1.831 | 0.624 | 6.25 | 0.0033 |
| Lymph node metastasis (positive) | 1.287 | 0.547 | 3.62 | 0.0187 |
| Subclassification of depth (sm3) | 0.678 | 0.623 | 1.97 | 0.2763 |
| Age (≤70) | 0.628 | 0.679 | 1.87 | 0.3555 |
| Length (≤3.6 cm) | 0.571 | 0.565 | 1.77 | 0.3128 |
| Location (upper) | 0.153 | 0.807 | 1.16 | 0.8499 |
| Differentiation of SCC[a] (poorly) | 0.054 | 0.564 | 1.05 | 0.9240 |

[a]SCC, squamous cell carcinoma.

**Table 3** Survival period and site of recurrence in cases with submucosal carcinoma of the oesophagus

| Case | Age | Sex | Survival period | Site of recurrence | Lymphatic invasion | Blood vessel invasion | Intramural metastasis | Lymph node recurrence |
|---|---|---|---|---|---|---|---|---|
| 1 | 77 | M | 4M | Lung | + | – | – | + |
| 2 | 51 | M | 9M | Surgical margin | + | – | – | + |
| 3 | 63 | M | 10M | Lung, bone | + | + | + | + |
| 4 | 59 | M | 1Y 1M | Mediastinal LN[a] | + | – | – | + |
| 5 | 56 | M | 1Y 6M | Liver | + | – | – | – |
| 6 | 76 | M | 1Y 7M | Surgical margin | – | – | – | + |
| 7 | 62 | M | 1Y10M | Mediastinal LN[a] | – | + | – | – |
| 8 | 69 | M | 2Y | Cervical LN[a] | + | – | – | + |
| 9 | 73 | M | 2Y 2M | Hilar LN[a] | – | + | – | – |
| 10 | 61 | M | 2Y 2M | Lung | – | + | + | – |
| 11 | 47 | M | 2Y11M | Abdominal LN[a] | + | – | – | – |
| 12 | 71 | M | 3Y 3M | Liver | – | – | – | – |
| 13 | 70 | M | 3Y 4M | Surgical margin | + | – | – | + |
| 14 | 58 | M | 4Y | Surgical margin | – | – | + | – |
| 15 | 60 | M | 6Y10M | Lung | – | – | – | + |

[a]Lymph node.

The long-term survival of patients with sm carcinoma of the oesophagus has been reported to be significantly poorer than those with intraepithelial or mucosal carcinoma (Sugimachi et al, 1989, 1993). Regarding the poor prognosis, the well-developed intramural lymphatic network of the oesophagus, especially in the sm layer, and consequently frequent lymph node involvement have been pointed out (Akiyama et al, 1994b). Therefore, a subtotal oesophagectomy with extended lymph node dissection, as is usually performed for advanced cancer, has been recommended (Yoshinaka et al, 1991; Kato et al, 1993; Watanabe et al, 1995).

© 2000 Cancer Research Campaign

Owing to these efforts, an improved survival rate has begun to be reported. The 5-year survival rate of patients with sm carcinoma was 74% in our series, which was as good as that noted in other recent reports in Japan (Yoshinaka et al, 1991; Kato et al, 1993; Watanabe et al, 1995). Nevertheless, there were more than a few patients who died of recurrent carcinoma after a curative oesophagectomy, as shown in Table 3. In these patients, the carcinoma has already spread beyond a surgically resectable area at the time of the operation, and it is thus impossible to cure them by radical surgery alone. The aim of this study was to clarify the factors concerning recurrence after a curative oesophagectomy for patients with sm carcinoma of the oesophagus in order to select the patients who had a high risk of recurrence. As a result, VI, IM and LN were proven to be significant prognostic factors by the Cox regression analysis.

Lymph node metastasis is known to be an important cause of death in cancer of the oesophagus. There have also been several reports on the prognostic significance of lymph node metastasis in superficial carcinoma of the oesophagus (Yoshinaka et al, 1991; Nishimaki et al, 1998; Matsubara et al, 1999). In this study, the survival of patients with LN was significantly poorer than that without, and LN was identified as one of the independent prognostic factors. On the other hand, although the prognostic significance of VI or IM in squamous cell carcinoma of the oesophagus has been reported previously (Takubo et al, 1990; Theunissen et al, 1991; Kuwano et al, 1994; Sarbia et al, 1995), few reports have shown the significance of them in sm carcinoma. In this study, we revealed that these factors were also independent prognostic factors. The question then arises about the rate of VI in sm carcinoma of the oesophagus that varies according to the articles. A higher rate was reported by Nishimaki et al (1994), 16 of 27 cases (59.3%), and by Nabeya and Nakata (1997), LVI in 701 of 1225 cases (57.2%) and BVI in 311 of 1225 cases (25.4%). While Sarbia et al reported that 12 cases (28.6%) with LVI and 4 cases (9.5%) with BVI were found in 42 cases with pT1/T2 carcinoma (1995). In our series, the rate was closer to the latter. Such variation is probably caused by differences in the criteria for determining VI. We have diagnosed all cases according to the above-described criteria and confirmed the usefulness of such criteria for predicting the prognosis.

Finally, we shall discuss the indication for adjuvant therapy after surgery. As shown in Figure 3A, the survival of Group I was significantly better than that of Group II, and 5-year survival rate of Group I patients was as good as that of mucosal cancer. However, the difference of the prognosis between Groups III and IV, shown in Figure 3B, was more significant than that between Groups I and II, and there were 14 of 40 patients (35.0%) who died of recurrent cancer within 5 years in Group II while there were 12 of 26 (46.2%) in Group IV. For reasons mentioned above, we may say that Group IV patients, i.e. patients with either VI or IM, are candidates for adjuvant therapy.

In conclusion, based on the above findings, VI, IM and LN are thus considered to be significant prognostic factors in patients with sm carcinoma of the oesophagus. In order to improve the prognosis of sm carcinoma of the oesophagus, we still need to identify the optimal adjuvant therapy for patients with either VI or IM positive sm carcinoma of the oesophagus.

### REFERENCES

Akiyama H, Tsurumaru M, Udagawa H and Kajiyama Y (1994a) Radical lymph node dissection for cancer of the thoracic esophagus. *Ann Surg* **220:** 364–373

Akiyama H, Tsurumaru M, Udagawa H and Kajiyama Y (1994b) Systemic lymph node dissection for esophageal cancer – effective or not? *Dis Esophagus* **7:** 2–13

Bogomoletz WV, Molas G and Gayet B (1989) Superficial squamous cell carcinoma of the esophagus: a report of 76 cases and review of literature. *Am J Surg Pathol* **3:** 535–546

Brodmerkel GJ (1971) Shiller's test: an aid in esophagoscopic diagnosis. *Gastroenterol* **60:** 813

Inoue T, Mori M, Shimono R, Kuwano H and Sugimachi K (1992) Vascular invasion of colorectal carcinoma readily visible with certain stains. *Dis Colon Rectum* **35:** 34–39.

Isono K, Sato H and Nakayama K (1991) Results of a nationwide study on the three-field lymph node dissection of esophageal cancer. *Oncology* **48:** 411–420

Japanese Society for Esophageal Diseases (1999) *Guidelines for the Clinical and Pathologic Studies on Carcinoma of the Esophagus* (the 9th edition).

Kato H, Tachimori Y, Mizobuchi S, Igaki H and Ochiai A (1993) Cervical, mediastinal, and abdominal lymph node dissection (three-field dissection) for superficial carcinoma of the thoracic esophagus. *Cancer* **72:** 2879–2882

Kitamura K, Ikebe M, Morita M, Matsuda H, Kuwano H and Sugimachi K (1993) The evaluation of submucosal carcinoma of the esophagus as a more advanced carcinoma. *Hepato-Gastroentel* **40:** 236–239

Kodama M and Kakegawa T (1998) Treatment of superficial cancer of the esophagus: a summary of responses to a questionnaire on superficial cancer of the esophagus in Japan. *Surgery* **123:** 432–439

Kuwano H, Watanabe M, Sadanaga N, Kamakura T, Nozoe T, Yasuda M, Mimori K, Mori M and Sugimachi K (1994) Univariate and multivariate analysis of the prognostic significance of discontinuous intramural metastasis in patients with esophageal cancer. *J Surg Oncol* **57:** 17–21

Lehnert T (1999) Multimodal therapy for squamous carcinoma of the oesophagus. *Br J Surg* **86:** 727–739

Nabeya K and Nakata Y (1997) Topic forum: early esophageal cancer. Extent of resection and lymphadenectomy in early squamous cell esophageal cancer. *Dis Esophagus* **10:** 159–161

Nishimaki T, Tanaka O, Suzuki T, Aizawa K, Hatakenaka K and Muto T (1994) Patterns of lymphatic spread in thoracic esophageal cancer. *Cancer* **74:** 4–11

Nishimaki T, Suzuki T, Kanda T, Obinata I, Komumai S and Hatakeyama K (1998) Extended radical esophagectomy for superficially invasive carcinoma of the esophagus. *Surgery* **125:** 142–147

Sarbia M, Porschen R, Borchard F, Horstmann O, Willers R and Gabbert HE (1995) Incidence and prognostic significance of vascular and neural invasion in squamous cell carcinoma of the esophagus. *Int J Cancer* **61:** 333–336

Schmidt LW, Dean PJ and Wilson RT (1986) Superficially invasive squamous cell carcinoma of the esophagus. *Gastroenterol* **91:** 1456–1461

Shimizu H, Kobori O and Shoji M (1983) Superficial carcinoma of the esophagus. *Gastroenterol Jpn* **18:** 409–416

Sugimachi K, Ohno S, Matsuda H, Mori M, Matsuoka H and Kuwano H (1989) Clinicopathologic study of early stage esophageal carcinoma. *Surgery* **105:** 706–710

Sugimachi K, Kitamura K, Matsuda H, Mori M, Kuwano H and Ide H (1991) Proposed new criteria for early carcinoma of the esophagus. *Surg Gynecol Obstet* **173:** 303–308

Sugimachi K, Ikebe M, Kitamura K, Toh Y, Matsuda H and Kuwano H (1993) Long-term results of esophagectomy for early esophageal carcinoma. *Hepato-Gastroenterol* **40:** 203–206

Takubo K, Sasashima K, Yamashita K, Tanaka Y and Fujita K (1990) Prognostic significance of intramural metastasis in patients with eesophageal carcinoma. *Cancer* **65:** 1816–1819

Theunissen PHMH, Borchard F and Poortvliet (1991) Histopathlogical evaluation of oesophageal carcinoma: the significance of venous invasion. *Br J Surg* **78:** 930–932

Watanabe H, Kato H, Tachimori Y, Kise Y, Nakanishi Y and Ochiai A (1995) Necessity of cervical lymph node dissection by retrospective analysis of submucosal cancer in mid- and lower thoracic esophagus. *Ann Thorac Cardiovasc Surg* **1:** 49–53

Yoshinaka H, Shimizu H, Fukumoto T and Baba M (1991) Superficial esophageal carcinoma: a clinicopathological review of 59 cases. *Am J Gastroenterol* **86:** 1413–1418

*© 2000 Cancer Research Campaign*
